# Intrauterine fetal growth restriction in sheep leads to sexually dimorphic programming of Preadipocytes' differentiation potential

**DOI:** 10.14814/phy2.70143

**Published:** 2024-12-03

**Authors:** Michell Goyal, Rosa I. Luna Ramirez, Sean W. Limesand, Ravi Goyal

**Affiliations:** ^1^ Departmet of Physiology University of Arizona Tucson Arizona USA; ^2^ School of Animal and Comparative Biomedical Sciences, College of Agriculture and Life Sciences University of Arizona Tucson Arizona USA; ^3^ Department of Obstetrics and Gynecology University of Arizona Tucson Arizona USA

**Keywords:** adipose, developmental origins hypothesis, DOHaD, epigenetics, fetal growth‐retardation, fetal programming, IUGR, RNAseq

## Abstract

Fetal growth restriction (FGR) is a risk factor for obesity in adult life. Importantly, growth‐restricted females are more prone to obesity than males. The mechanisms involved in this sexually dimorphic programming are not known. Previously, we have demonstrated that ambient hyperthermia (40°C) led to placental insufficiency and significant FGR, and the perirenal adipose tissue undergoes sexually dimorphic gene expression. We demonstrated that males undergo significant changes in gene expression with growth restriction. This was not the case in females. We have also demonstrated that the isolated preadipocytes from male FGR (MFGR) have reduced differentiation potential compared to control males & females and female FGR (FFGR). Thus, we hypothesized that growth restriction differentially programs gene expression and genetic pathways in perirenal preadipocytes, which reduces their differentiation potential in male fetuses in a sexually dimorphic manner. We created FGR by exposing pregnant sheep to ambient hyperthermia. After isolating preadipocytes from perirenal adipose tissue, we differentiated them following published protocols. We examined the gene expression before and after differentiation from control male, control female, MFGR, and FFGR female. We also compared our data with other published studies in mouse and human preadipocytes. Our results demonstrate that a set of 21 genes altered with preadipocyte differentiation to mature adipocytes is common in adipose tissue from both sexes, humans, mice, and sheep, at different organismal ages (embryonic, fetal, and adult) and different sites (subcutaneous inguinal, pancreatic, perirenal). We also demonstrate that female FFGR fetuses demonstrate all these 21 genes altered similar to control males and females; however, MFGR fetuses have six genes (Dgat2, Fabp4, Lipe, Lrrfip1, Spred3, and Thrsp) that are not changed with preadipocyte differentiation to mature adipocyte. These genes may be responsible for reduced differentiation potential and obesity in FGR males compared to FGR females. Another important finding of the present study is that Lrrfip1, known to be associated with obesity, was upregulated with FGR and requires further investigation. Overall, our studies provide several target genes that may play a crucial role in reducing the risk of MFGR for obesity.

## 1 INTRODUCTION

Obesity is now considered an epidemic that has affected every nation and society on earth. The developmental origins of health and disease (DOHaD) hypothesis have implicated fetal growth restriction (FGR) as an important risk factor in developing obesity in later life (Goyal & Longo, 2013; Hanson & Gluckman, 2008). The worldwide FGR affects 7%–10% of pregnancies or about 20.5 million infants annually (de Onis et al., 1998; Reece, 2008). Our previous studies in mice have confirmed that low birth weight may cause dysregulation of glucose metabolism, obesity, and hypertension in later life (Goyal et al., 2015; Goyal & Longo, 2013). Recent evidence suggests that adipose tissue has a population of preadipocytes (also known as adipose‐derived stem cells), which can differentiate into mature adipocytes and cells of several lineages (Blomberg et al., 2023). These preadipocytes can cause hyperplasia and increase adipose tissue mass, leading to obesity (Kulenkampff & Wolfrum, 2019).

The thrifty phenotype hypothesis explains that the in‐utero environment of reduced nutrient availability prepares the fetus for the predicted outside environment of nutrient scarcity (Hales & Barker, 2001). Studies indicate that a fetus is “programmed” to live under thrifty conditions. Suppose adequate nutrition is available to the newborn postnatally. In that case, excess nutrition, such as fat, starts accumulating for future nutritional starvation, leading to rapid catch‐up growth, a risk factor for obesity (Crume et al., 2014; Goyal & Longo, 2013).

Moreover, it is well established that Female FGR (FFGR) are more prone to obesity than male FGR (MFGR) (Dearden et al., 2018). Our previous work demonstrated that ambient hyperthermia induces significant FGR (Blomberg et al., 2023). Additionally, we showed that gene expression in perirenal adipose tissue exhibits sexually dimorphic changes in response to growth restriction, with marked differences between males and females compared to their respective controls. However, as adipose tissue consists of various cell types and blood vessels, it was essential to isolate preadipocytes to better understand the specific effects of FGR. Our findings revealed that preadipocytes from MFGR have reduced differentiation potential compared to those from female FFGR. Interestingly, FFGR offspring are more susceptible to obesity than their male counterparts. To further elucidate the underlying mechanisms, we examined gene expression changes during the differentiation of primary preadipocytes into adipocytes from control male (MC), control female (FC), MFGR, and FFGR. Our goal was to identify specific gene signatures that may confer relative protection against obesity in MFGR compared to FFGR, providing insight into the sexually dimorphic programming of adipose tissue in FGR. Our previous report demonstrates that adipose tissue from FFGR shows a gene expression profile different from MFGR (Blomberg et al., 2023); the current study was done on preadipocyte cells isolated from adipose tissue and differentiated to mature adipocytes. Thus, the present study further investigated the underlying molecular pathways involved in differential programming of males and females to obesity in response to maternal stress.

To determine the molecules and pathways involved in reduced differentiation potential, we hypothesized that growth restriction differentially programs gene expression and genetic pathways in perirenal preadipocytes, which reduces differentiation potential in male fetuses in a sexually dimorphic manner. We speculate that these differential gene regulations in males and females with growth restriction make the females more prone to obesity in adult life.

## 2 METHODS

The University of Arizona's Institutional Animal Care and Use Committee (IACUC) reviewed and approved all animal studies.

### 2.1 FGR model

Crossbred Columbia‐Rambouillet ewes carrying singleton pregnancies were used in this study. We used ambient hyperthermia to produce placental insufficiency and restrict in‐utero fetal growth as described previously (Blomberg et al., 2023). In this model of FGR, we exposed pregnant ewes to elevated ambient temperatures (40°C for 12 h; 35°C for 12 h; dew point 22°C) from 38 ± 1 to 87 ± 1 days of gestation (total gestation in sheep is ~145 days). Control fetuses were from ewes maintained at 22 ± 1°C and pair‐fed to the hyperthermic ewe group's average ad libitum feed intake. The sheep feed was obtained from Maid Rite Feeds, Willcox, AZ, USA, and consisted of standard alfalfa pellets supplemented with trace minerals (Code #1131‐Z). All ewes are given ad libitum access to water and salt. All studies were conducted on the four study groups—near‐term MC fetuses, near‐term control female fetuses (FC), near‐term FGR male fetuses (MFGR), and near‐term FGR female fetuses (FFGR).

### 2.2 Preadipocyte isolation

We isolated perirenal fat from all four study groups to examine functional differences and changes in the “programming” of preadipocyte cells. At term, both the ewe and fetus were euthanized with an overdose of sodium pentobarbital (86 mg/kg) and phenytoin sodium (11 mg/kg, Euthasol; Virbac Animal Health) given IV. As published previously (Blomberg et al., 2023)., the perirenal fat was carefully dissected after the removal of the kidneys and snap‐frozen in liquid nitrogen, and stored at −80°C until further analysis. Perirenal fat is a visceral subtype with well‐defined boundaries and is covered with a perirenal fascia (Gerota's fascia), which makes it easier to isolate and conduct weight measurements.

### 2.3 Whole transcriptomic analysis

We have described this method in detail in our previous publication (Blomberg et al., 2023). Briefly, preadipocytes and differentiated cell pellets were thawed and RNA was isolated using Trizol. The RNA was further purified by Zymo Purelink RNA columns. The obtained RNA samples were measured for quantity (ng/mL) and purity on Nanodrop and Qubit (broad‐range) according to 260/280 nucleic acid absorbance ratio before sending the University of Arizona Genetics Core Facility for sequencing, where RNA was further checked for quality and quantity with an Advanced Analytics Fragment Analyzer (High Sensitivity RNA Analysis Kit—Catalog # DNF‐491/User Guide DNF‐491‐2014AUG13) and quantity with determined with a Qubit RNA quantification kit (Qubit® RNA HS Assay Kit—Catalog # Q32852).

Once quality and quantity were validated, a library was constructed from samples using a Swift RNA Library Kit—(Catalog # R1024/Swift Protocol version 3.0) and Swift Dual Combinatorial Indexing Kit—(Catalog # X8096). Upon constructing the library, the Advanced Analytics Fragment Analyzer with the High Sensitivity NGS Analysis Kit—(Catalog # DNF‐486 / User Guide DNF‐486‐2014MAR10) determined the average fragment size. Quantity was evaluated with an Illumina Universal Adaptor‐specific qPCR kit, the Kapa Library Quantification Kit for Illumina NGS—(Catalog # KK4824 / KAPA Library Quantification Technical Guide—AUG2014). After completing the final library QC, samples were equimolar‐pooled and clustered for sequencing on the NextSeq500 machine. The sequencing run was performed using Illumina NextSeq500 run chemistry (NextSeq 500/550 High Output v2 kit 150 cycles‐Catalog FC‐404‐2002).

### 2.4 Comparative RNAseq data analysis

We searched GEO Datasets using the terms Preadipocytes and differentiation and “high throughput sequencing” [platform] to find other RNAseq studies. Sequence data quality was validated for RNAseq analysis using FastQC (v0.12.1). Sequences with average Phred scores below 34 were discarded. Fully annotated genome indices were generated for sheep (Oar_rambouillet_v1.0), mice (GRCm39), and humans (GRCh38) using ENSEMBL genome browser and aligned with the sequencing data using Salmon (Patro et al., 2017). Differential gene expression datasets were created using the DESEQ2 (version 3.19) in R‐studio. Our DESEQ2 analysis provided log2tranformed expression data normalized to estimate size factors using the “median ratio method” (log2FC) and their respective FDR‐adjusted p‐values (p‐adj). Pathway analysis was conducted with iDEP 2.01 web‐based applications (Ge et al., 2018). During preprocessing, the genes with less than 20 counts per million in 3 or more libraries were discarded from further processing, and counts were normalized using regularized log transformation. The distribution of the transformed data box and density plots are provided in Figure S1. Enrichment analysis for differentially expressed genes was conducted using a false discovery rate (FDR) cutoff of 0.05 and a minimum fold change of 1.5. Genes altered were matched with the known Kyoto encyclopedia of genes and genomes (KEGG) pathways (Kanehisa & Goto, 2000), and gene set enrichment analysis (GSEA) was conducted as published (Subramanian et al., 2005). Generally applicable gene‐set enrichment (GAGE) was also conducted to determine the biological relevance of the regulatory mechanism (Luo et al., 2009). All the data is deposited on the GEO database (accession No. GSE278762).

### 2.5 Statistical analysis

The statistical analysis was conducted using DESEQ2 on R‐Program (Version 4.3.2).

## 3 RESULTS

### 3.1 RNAseq analysis of the preadipocytes isolated from the perirenal adipose tissue

**FIGURE 1:**
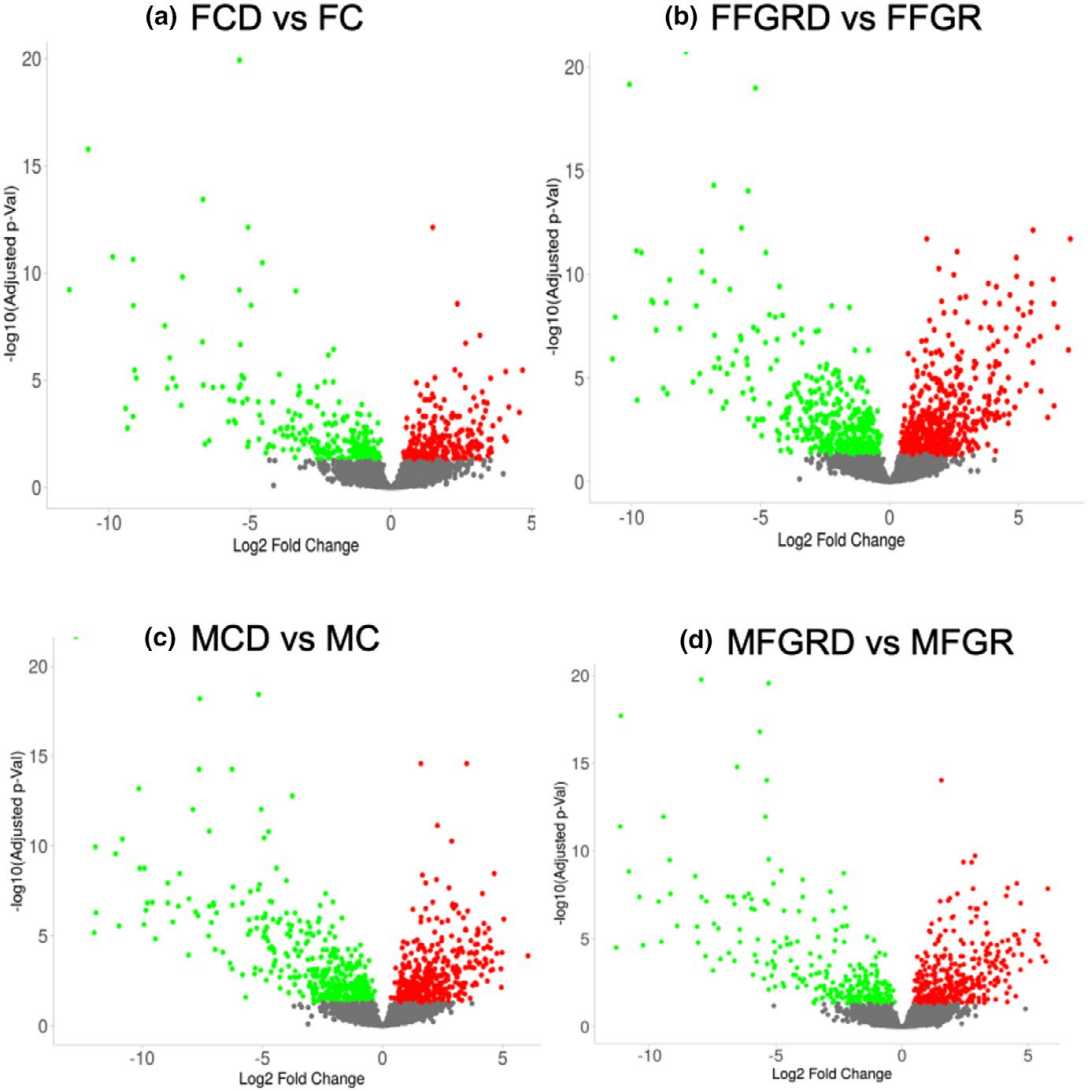
Demonstrate the volcano plots of differentially regulated genes following differentiation of preadipocytes to adipocytes at the Log2 Fold change of >1.5 and adjusted p‐value <0.05. (a) Control fetal female differentiated (FCD) adipocytes versus control fetal female (FC) preadipocytes. (b) Female fetal growth‐restricted differentiated adipocytes (FFGRD) versus female fetal growth‐restricted preadipocytes (FFGR). (c) Control fetal male differentiated (MCD) adipocytes versus control fetal male (MC) preadipocytes. (d) Male fetal growth‐restricted differentiated adipocytes (MFGRD) versus male fetal growth‐restricted preadipocytes (FFGR).

**FIGURE 2:**
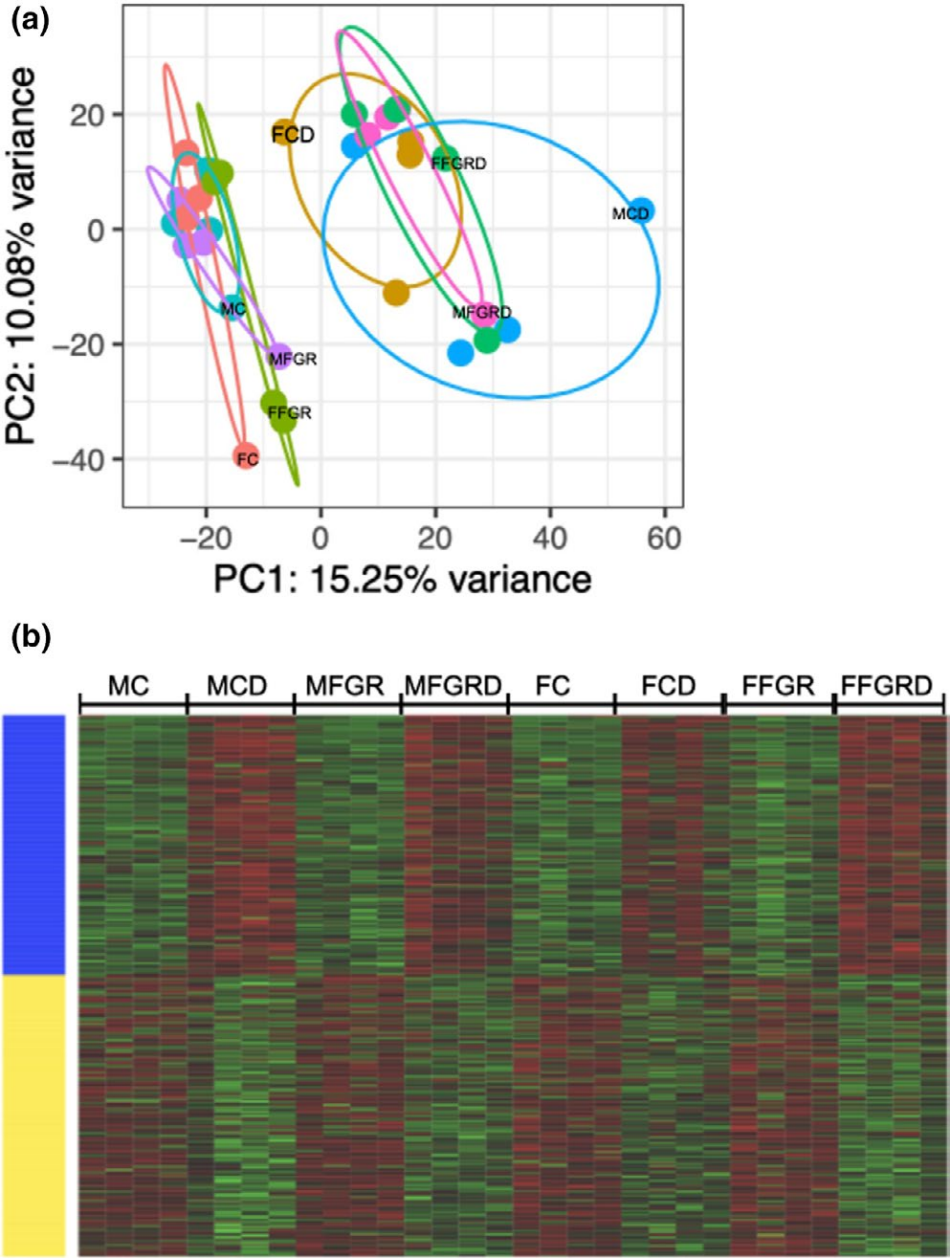
Shows the differential clustering of the genes from the eight study groups. (a) The Principal Component Analysis. (b) The heat map with red shows the upregulated genes, and green shows the downregulated genes. MC, male control preadipocytes; FC, female control preadipocytes; MCD, male control differentiated adipocytes; FCD, female control differentiated adipocytes; MFGR, male fetal growth restricted preadipocytes; FFGR, female fetal growth restricted preadipocytes; MFGRD, male fetal growth restricted differentiated adipocytes; FFGRD, female fetal growth restricted differentiated adipocytes.

The results demonstrate a strong effect of cell differentiation on gene expression (Table 1 and Figure 1). The groups clustered together into preadipocytes and differentiated cells (Figure 2a). The hierarchical clustering demonstrates a similar trend (Figure 2b).

**TABLE 1:** Shows the differentially regulated genes (fold change >1.5 and adjusted *p*‐value (FDR) <0.05 in various study groups.

Comparison groups	Upregulated	Downregulated	Total
MC vs. FC	9	14	23
MCD vs. FCD	20	24	44
MFGR vs. FFGR	28	30	58
MFGRD vs. FFGRD	7	11	18
MCD vs. MC	879	757	1636
FCD vs. FC	270	318	588
MFGRD vs. MFGR	431	639	1070
FFGRD vs. FFGR	535	653	1188

Abbreviations: FC, female control preadipocytes; FCD, female control differentiated adipocytes; FFGR, female fetal growth restricted preadipocytes; FFGRD, female fetal growth restricted differentiated adipocytes; MC, male control preadipocytes; MCD, male control differentiated adipocytes; MFGR, male fetal growth restricted preadipocytes; MFGRD, male fetal growth restricted differentiated adipocytes.

### 3.2 Effect of sex on gene expression in preadipocytes differentiation to mature adipocytes

**FIGURE 3:**
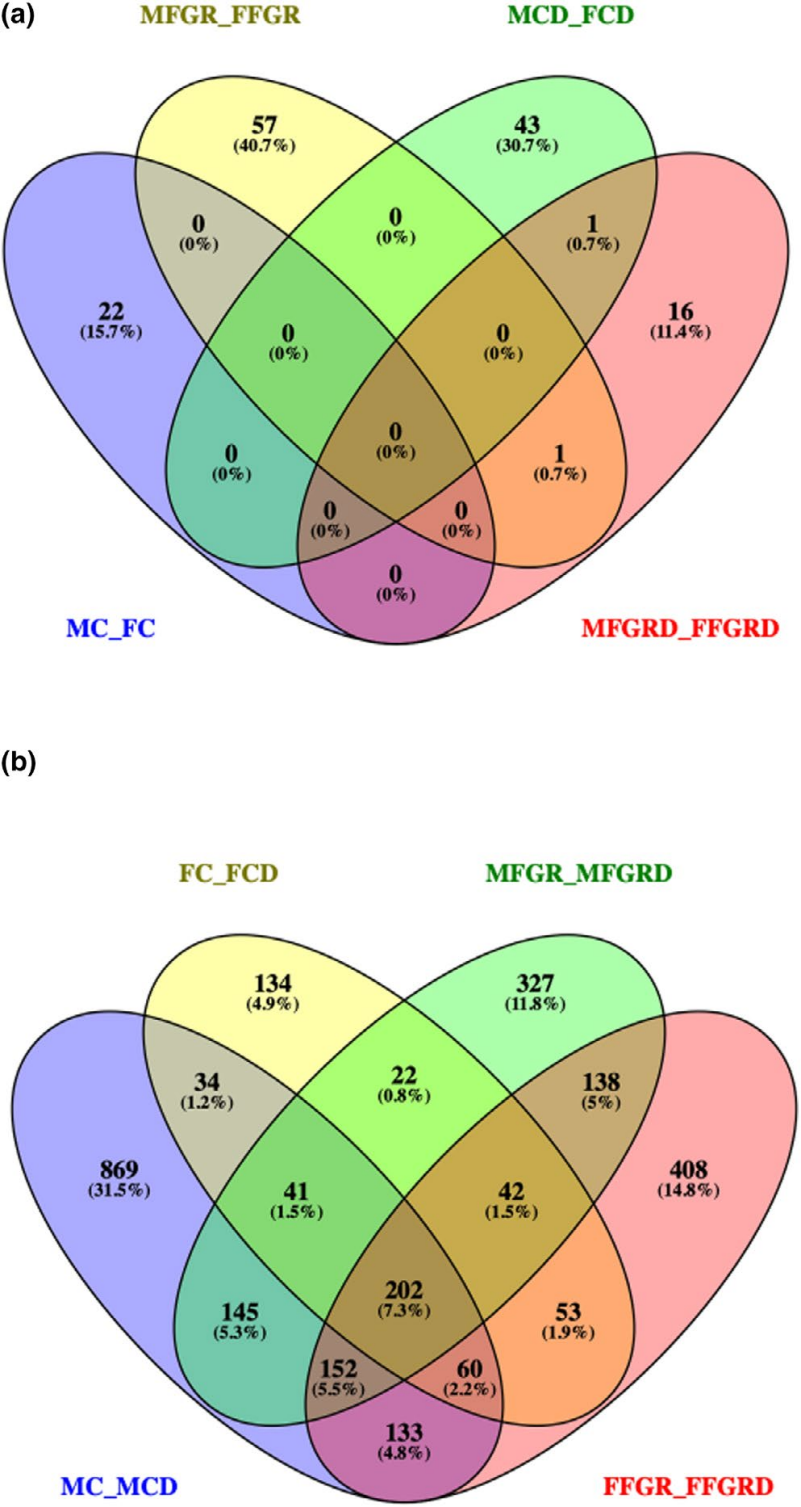
Shows the Venn Diagram of the commonly altered gene (a) Effect of sex on gene regulation. (b) Effect of preadipocyte to mature adipocyte differentiation on gene expression. MC, male control preadipocytes; FC, Female control preadipocytes; MCD, male control differentiated adipocytes; FCD, female control differentiated adipocytes; MFGR, male fetal growth restricted preadipocytes; FFGR, female fetal growth restricted preadipocytes; MFGRD, male fetal growth restricted differentiated adipocytes; FFGRD, female fetal growth restricted differentiated adipocytes.

The overall changes in gene expression with sex are listed in Table 1. Specifically, 73 genes were differentially expressed in the preadipocytes from MC and FC. Of these, nine genes were upregulated and 14 downregulated in females compared to males (Table S1). To further elucidate the effect of sex on differential gene expression, we compared differentiated preadipocytes from male (MCD) and female (FCD) fetuses. We observed that 44 genes were differentially expressed in differentiated male adipocytes compared to those from female fetuses (Table S2). When we compared gene expression in preadipocytes from MFGR with FFGR, we observed that 58 genes were differentially expressed (Table S3). We also compared alterations in gene expression between differentiated adipocytes from the growth‐restricted male (MFGRD) and female (FFGRD) and observed that 18 genes were significantly differentially expressed (Table S4). Surprisingly, only one gene was the same between the altered gene in differentiated adipocytes from preadipocytes in control and growth‐restricted fetuses (Figure 3a).

### 3.3 Effect of growth restriction on gene expression in preadipocytes following differentiation to mature adipocytes

The project aimed to identify genes differentially expressed in male and female control and growth‐restricted fetus preadipocytes with differentiation to mature adipocytes. We observed a strong effect of preadipocyte differentiation on gene expression (Figure 2a,b). In MCs with preadipocyte to mature adipocyte differentiation, there were 1636 genes differentially regulated (Table S5), whereas in females, only 588 genes were differentially regulated (Table S6). However, with growth restriction, 1069 genes in males (Table S7) and 1188 genes (Table S8) in females were differentially regulated on converting preadipocytes to mature adipocytes. Thus, there was a significant decrease in the number of genes altered with growth restriction in males (gene altered reduced from 1636% to 1069% ~ 35% reduction with cell differentiation. In contrast, the number of differentially altered genes increased in females from 588 to 1188. Thus, sex strongly affected differential gene regulation with preadipocyte differentiation.

In MCs and females, 337 common transcripts of 319 genes were differentially regulated with preadipocyte differentiation to mature adipocytes (Table S9). However, in growth‐restricted males and females, 534 transcripts of 486 genes were commonly differentially regulated with preadipocyte differentiation to mature adipocytes (Table S10). On comparing changes in gene expression in all four groups with differentiation, we observed 202 transcripts of 192 genes altered commonly in all four groups (irrespective of FGR) following the differentiation of preadipocytes to mature adipocytes (Figure 3b and Table S11).

Previously, we have demonstrated that the isolated preadipocytes from MFGR have reduced differentiation potential compared to MCs & females and FFGR. We observed that the three groups (MC, FC, and FFGR) with higher differentiation potential than MFGR had 262 commonly differentiated transcripts of 249 genes. Thus, they had 60 genes extra differentially expressed than the genes common to all four groups (Table S12). On importance, following differentiation, the three groups, MC, FC, and FFGR, have upregulated mature adipocyte markers adiponectin and FABP4. However, MFGR only had adiponectin upregulated, and FABP4 was not. Thus, there appear to be fundamental changes in male fetuses' adipose tissue regulation with growth restriction.

### 3.4 Comparison of the current data with other studies

We searched GEO Datasets using the terms preadipocytes and differentiation and “high throughput sequencing” [platform] to find other RNAseq studies. Our initial search identified 16 unique studies. The studies that did not have at least three control and three differentiated samples without treatment were excluded from the comparison. Following this, we identified six individual studies with the keywords mentioned above. None of the studies were done on preadipocytes from fetal lambs. One study did not provide raw data; another only examined microRNA, and both were excluded. For the four other studies (Table 2), raw data was downloaded and processed through the same pipeline as the original data. We observed that a total of 66 genes were common among the differentially regulated genes in all four studies (Table S13). Of these, 33 were upregulated, and 33 were downregulated. Notably, mature adipocyte markers genes such as adiponectin were not present among these genes. One of the studies was conducted after adding differentiation media for only 2 days. Others and we have shown that differentiating preadipocytes to mature adipocytes takes more than 5 days. Thus, we removed this study with only 2 days of differentiation protocol and determined the common genes in the other three studies with complete differentiation protocols. We observed all the markers of mature adipocytes, such as adiponectin, FABP4, Cidea, etc., to be upregulated in all three studies. Of note, 243 genes were commonly differentiated in the three studies (Table S14). Two of these studies were conducted on mouse preadipocytes and one on human preadipocytes.

**TABLE 2:** Enlists the studies and SRA downloaded from the GEO database for comparative analysis.

Accession No.	Description	Species	SRA used
GSE145452	3 T3‐L1 Preadipocytes control and differentiated for 2 days.	Mouse	SRR11100850 SRR11100851 SRR11100852 SRR11100856 SRR11100857 SRR11100858
GSE169514	Pancreatic preadipocytes were differentiated for 14 days.	Human	SRR14060251 SRR14060252 SRR14060253 SRR14060254 SRR14060255 SRR14060256 SRR14060257 SRR14060258 SRR14060264 SRR14060265 SRR14060267 SRR14060268 SRR14060270 SRR14060271 SRR14060272 SRR14060273
GSE193463	Subcutaneous inguinal preadipocytes differentiated for 8 days.	Mouse	SRR17518441 SRR17518442 SRR17518443 SRR17518447 SRR17518448 SRR17518449 SRR17518453 SRR17518454 SRR17518455
GSE201450	3 T3‐L1 preadipocytes for 9 days.	Mouse	SRR18912033 SRR18912034 SRR18912035 SRR18912024 SRR18912025 SRR18912026

Next, we identified the common genes in our studies (MC/MCD, MFGR/MFGRD, FC/FCD, FFGR/FFGRD) with the four studies from GEO datasets. We observed only four common genes in all GSE datasets with our studies. However, this dataset included studies with growth restriction and only 2 days of differentiation (early initiation) till complete differentiation. These four genes were upregulated with differentiation and were FKBP5 (ENSOART00020035500), ACSL1 (ENSOART00020021450), PLIN4 (ENSOART00020021654) and ZBTB16 (ENSOART00020035665).

We further compared commonly differentiated genes from our complete datasets (Table S11) with the commonly regulated genes from the three GSE downloaded studies, which studied differentiation till the late stage (data in Table S14) and observed a total of 12 genes (9 upregulated and 3 downregulated in all groups) differentially regulated in the three species (mouse, human, and sheep), different ages (fetal to adult), and different nutritional conditions (control to growth restricted) with differentiation of preadipocyte to mature adipocyte (Table 3).

**TABLE 3:** Enlists the common genes altered in all four study groups and the three GEO datasets.

Gene name	MCD/MC Fold_Change	FCD/FC Fold_Change	MFGRD/MFGR Fold_Change	FFGRD/FFGR Fold_Change	GSE169514‐Fold_Change	GSE193463‐Fold_Change	GSE201450‐Fold_Change
Klb	3869.02	241.04	1299.03	1662.77	44.61	196.30	212.78
Adipoq	1147.91	80.22	293.32	54.25	12508.15	522.97	308881.33
Plin4	265.10	226.97	32.10	557.28	38543.21	23.00	8.35
Zbtb16	199.79	1723.21	2232.69	1149.48	29.33	5275.86	18.74
Abcd2	54.95	30.89	198.14	232.79	36.19	7.74	21.24
Pdk4	47.37	23.69	97.47	45.21	4.21	6.85	2.03
Cebpa	45.13	8.56	23.64	24.55	59.98	3.66	22.71
Fkbp5	31.85	42.28	37.20	37.45	4.82	7.32	2.17
Acsl1	31.65	11.36	10.40	39.52	5.93	10.15	215.94
Pls3	−4.32	−2.90	−4.00	−5.87	−1.45	−1.53	−2.11
Nexn	−5.56	−6.91	−11.06	−9.91	−1.66	−3.02	−7.46
Postn	−14.44	−18.19	−11.09	−31.64	−2.74	−2.35	−14.47

Abbreviations: FC, female control preadipocytes; FCD, female control differentiated adipocytes; FFGR, female fetal growth restricted preadipocytes; FFGRD, female fetal growth restricted differentiated adipocytes; MC, male control preadipocytes; MCD, male control differentiated adipocytes; MFGR, male fetal growth restricted preadipocytes; MFGRD, male fetal growth restricted differentiated adipocytes.

Furthermore, we compared the commonly differentiated genes in our controls (MC/MCD and FC/FCD) with the three GSE downloaded studied and observed a total of 21 genes (14 upregulated and 7 downregulated in all groups) differentially regulated in the three species (mouse, human, and sheep) with differentiation of preadipocyte to mature adipocyte (Table 4). Of importance, when we compared the gene expression in the 3 GSE studies with 3 of our studies (MC/MCD, FC/FCD, FFGR/FFGRD), the gene‐altered were the same as shown in Table 4.

**TABLE 4:** Enlists the common genes altered in control study groups and the three GEO datasets.

Gene name	MCD/MC Fold_Change	FCD/FC Fold_Change	GSE169514‐Fold_Change	GSE193463‐Fold_Change	GSE201450‐Fold_Change
Fabp4	6928.63	568.18	7062.54	65.12	1369.70
Thrsp	3894.99	562.05	1689.18	3863.52	1151.43
Klb	3869.02	241.04	44.61	196.30	212.78
Adipoq	1147.91	80.22	12508.15	522.97	308881.33
Dgat2	417.70	351.42	12.54	2.79	46.23
Lipe	376.48	40.44	60.39	9.50	103.08
Plin4	265.10	226.97	38543.21	23.00	8.35
Zbtb16	199.79	1723.21	29.33	5275.86	18.74
Plin1	88.58	61.15	190.73	76.12	13975.33
Abcd2	54.95	30.89	36.19	7.74	21.24
Pdk4	47.37	23.69	4.21	6.85	2.03
Cebpa	45.13	8.56	59.98	3.66	22.71
Fkbp5	31.85	42.28	4.82	7.32	2.17
Acsl1	31.65	11.36	5.93	10.15	215.94
Lrrfip1	−2.21	−3.94	−1.52	−1.63	−1.14
Tpm2	−3.32	−4.87	−2.08	−2.22	−2.97
Pls3	−4.32	−2.90	−1.45	−1.53	−2.11
Nexn	−5.56	−6.91	−1.66	−3.02	−7.46
Lmo7	−8.61	−3.75	−1.99	−4.83	−2.93
Postn	−14.44	−18.19	−2.74	−2.35	−14.47
Spred3	−18.81	−25.23	−2.96	−2.53	−2.35

Abbreviations: FC, female control preadipocytes; FCD, female control differentiated adipocytes; FFGR, female fetal growth restricted preadipocytes; FFGRD, female fetal growth restricted differentiated adipocytes; MC, male control preadipocytes; MCD, male control differentiated adipocytes; MFGR, male fetal growth restricted preadipocytes; MFGRD, male fetal growth restricted differentiated adipocytes.

Lastly, we compared the gene expression in the 3 GSE studies with 3 of our studies (MC/MCD, FC/FCD, FFGR/FFGRD; Table 4) with those altered in all study groups, including MFGR/MFGRD (Table 3), we observed six genes were not altered in MFGR/MFGRD as compared to all other groups. These were Dgat2, Fabp4, Lipe, Lrrfip1, Spred3, and Thrsp. These genes might be responsible for significantly reduced differentiation potential in MFGR preadipocytes.

### 3.5 Pathway analysis

The pathway analysis demonstrated significant changes in pathways altered due to FGR and differentiation. Notably, pathways involved in lipid metabolism, including the diacylglycerol metabolic pathway and acylglycerol‐O‐acetyl transferase activity pathways, were downregulated by following perirenal preadipocyte differentiation in all four groups (Table 5).

**TABLE 5:** Enlists the major pathways identified by the differential gene regulation. Orange color indicates common genes in all the study groups. Green color highlights the lipid metabolism pathways altered in control males and females following differentiation of preadipocytes. Pink color highlights the common pathways in males and females. Blue color highlights the lipid metabolism pathways upregulated in FFGR as compared to MFGR. Yellow color highlights the lipid metabolism pathways upregulated in MFGRs as compared to FFGR.

Direction	GSEA analysis: FC vs. FCD pathways	NES	Genes	Adj.Pval	
Down	GO:0015294 solute: cation symporter activity	−0.8762	13	7.00E‐03	Common in all four study groups
	GO:0016411 acylglycerol O‐acyltransferase activity	−0.9087	9	1.80E‐02	Lipid metabolism pathways different in control males vs. females
	GO:0032634 interleukin‐5 production	−0.9504	5	3.20E‐02	Pathways common in males and females
	GO:0032674 regulation of interleukin‐5 production	−0.9504	5	3.20E‐02	Lipid metabolism pathways different in FFGR as compared to MFGR
	GO:0046339 diacylglycerol metabolic process	−0.9044	8	3.20E‐02	Lipid metabolism pathways different in MFGR as compared to FFGR
	GO:0034383 low‐density lipoprotein particle clearance	−0.8983	8	3.90E‐02	
	GO:0016597 amino acid binding	−0.8959	8	3.90E‐02	
	GO:0038065 collagen‐activated signaling pathway	−0.9044	7	6.50E‐02	
	GO:0070391 response to lipoteichoic acid	−0.9266	5	8.40E‐02	
	GO:0071223 cellular response to lipoteichoic acid	−0.9266	5	8.40E‐02	
	GO:0015296 anion: cation symporter activity	−0.9125	6	1.00E‐01	
Up	Path:oas05150 Staphylococcus aureus infection	0.9127	10	7.60E‐04	
	GO:2000345 regulation of hepatocyte proliferation	0.9444	5	2.00E‐02	
	GO:0034104 negative regulation of tissue remodeling	0.9233	6	2.20E‐02	
	GO:0031954 positive regulation of protein autophosphorylation	0.8937	7	3.20E‐02	
	GO:0016327 apicolateral plasma membrane	0.9248	5	4.10E‐02	
	GO:0016339 calcium‐dependent cell–cell adhesion via plasma membrane cell adhesion molecules	0.9216	5	4.80E‐02	
	GO:0005614 interstitial matrix	0.9119	5	6.90E‐02	
	GO:0008009 chemokine activity	0.9104	5	7.10E‐02	
	GO:0017080 sodium channel regulator activity	0.8832	6	7.50E‐02	

Direction	GSEA analysis: FFGR vs. FFGRD Pathways	NES	Genes	adj.Pval
Down	Path:oas04923 Regulation of lipolysis in adipocytes	−0.8563	25	3.30E‐05
	GO:0046460 neutral lipid biosynthetic process	−0.849	17	1.10E‐03
	GO:0046463 acylglycerol biosynthetic process	−0.849	17	1.10E‐03
	GO:0016411 acylglycerol O‐acyltransferase activity	−0.9324	9	2.30E‐03

	GO:0019432 triglyceride biosynthetic process	−0.8555	13	1.10E‐02
	GO:0010226 response to lithium ion	−0.9469	5	2.70E‐02
	GO:0071285 cellular response to lithium ion	−0.9469	5	2.70E‐02
	GO:0046339 diacylglycerol metabolic process	−0.8986	8	2.80E‐02
	GO:0015296 anion: cation symporter activity	−0.9034	6	5.90E‐02
GO:0034383 low‐density lipoprotein particle clearance	−0.8672	8	7.50E‐02
Up	Path:oas05150 Staphylococcus aureus infection	0.8787	10	3.50E‐03
	GO:2000345 regulation of hepatocyte proliferation	0.962	5	4.20E‐03
	GO:0072574 hepatocyte proliferation	0.9009	7	2.40E‐02
	GO:0072575 epithelial cell proliferation involved in liver morphogenesis	0.9009	7	2.40E‐02
	GO:0042249 establishment of planar polarity of embryonic epithelium	0.8925	6	4.60E‐02
	GO:0090177 establishment of planar polarity involved in neural tube closure	0.8925	6	4.60E‐02
	GO:0090280 positive regulation of calcium ion import	0.891	6	4.80E‐02
	GO:0016339 calcium‐dependent cell–cell adhesion via plasma membrane cell adhesion molecules	0.9161	5	6.10E‐02
	GO:0048251 elastic fiber assembly	0.8693	6	8.40E‐02
	GO:0034104 negative regulation of tissue remodeling	0.8667	6	9.80E‐02

Direction	GSEA analysis: MC vs. MCD Pathways	NES	Genes	adj.Pval
Down	GO:0016411 acylglycerol O‐acyltransferase activity	−0.9478	9	3.10E‐04
	GO:0034383 low‐density lipoprotein particle clearance	−0.9419	8	1.10E‐03
	GO:0019433 triglyceride catabolic process	−0.9601	5	1.10E‐02
	GO:0046339 diacylglycerol metabolic process	−0.9	8	1.60E‐02
	GO:0010226 response to lithium ion	−0.944	5	2.50E‐02
	GO:0071285 cellular response to lithium ion	−0.944	5	2.50E‐02
	GO:0034114 regulation of heterotypic cell–cell adhesion	−0.8964	7	3.30E‐02
	GO:0034115 negative regulation of heterotypic cell–cell adhesion	−0.9339	5	5.90E‐02
	GO:0070587 regulation of cell–cell adhesion involved in gastrulation	−0.9339	5	5.90E‐02
	GO:0070586 cell–cell adhesion involved in gastrulation	−0.9057	6	6.00E‐02
	GO:0010889 regulation of sequestering of triglyceride	−0.9048	6	6.00E‐02
	GO:0045603 positive regulation of endothelial cell differentiation	−0.9174	5	7.40E‐02
	GO:0002178 palmitoyltransferase complex	−0.9134	5	8.20E‐02
	GO:0004806 triglyceride lipase activity	−0.9115	5	8.20E‐02
	GO:0006244 pyrimidine nucleotide catabolic process	−0.9057	5	9.30E‐02
Up	Path:oas05150 Staphylococcus aureus infection	0.9481	10	4.60E‐06
	GO:0034104 negative regulation of tissue remodeling	0.9601	6	5.20E‐04
	GO:0048251 elastic fiber assembly	0.9382	6	3.20E‐03
	GO:0060768 regulation of epithelial cell proliferation involved in prostate gland development	0.9458	5	7.80E‐03
	GO:0060346 bone trabecula formation	0.917	5	2.40E‐02

Direction	GSEA analysis: MFGR vs. MFGRD Pathways	NES	Genes	adj.Pval
Down	GO:0010226 response to lithium ion	−0.9723	5	1.10E‐02
	GO:0071285 cellular response to lithium ion	−0.9723	5	1.10E‐02
	GO:0016411 acylglycerol O‐acyltransferase activity	−0.9244	9	1.50E‐02
	GO:0098644 complex of collagen trimers	−0.8752	12	2.70E‐02
	GO:0046339 diacylglycerol metabolic process	−0.893	8	9.40E‐02
	GO:0060613 fat pad development	−0.9396	5	9.60E‐02
Up	Path:oas05150 Staphylococcus aureus infection	0.9019	10	5.10E‐03
	GO:0042379 chemokine receptor binding	0.8765	9	1.50E‐02
	GO:2000345 regulation of hepatocyte proliferation	0.9515	5	1.90E‐02
	GO:0072574 hepatocyte proliferation	0.9061	7	2.40E‐02
	GO:0072575 epithelial cell proliferation involved in liver morphogenesis	0.9061	7	2.40E‐02
	GO:0051797 regulation of hair follicle development	0.9034	7	2.70E‐02
	GO:0008009 chemokine activity	0.9356	5	4.30E‐02
	GO:0017080 sodium channel regulator activity	0.908	6	4.70E‐02
	GO:0070141 response to UV‐A	0.9303	5	5.00E‐02
	GO:0016327 apicolateral plasma membrane	0.9296	5	5.10E‐02
	GO:0021756 striatum development	0.8795	7	6.40E‐02
	GO:0048251 elastic fiber assembly	0.8917	6	8.10E‐02
	GO:0016339 calcium‐dependent cell–cell adhesion via plasma membrane cell adhesion molecules	0.9111	5	8.40E‐02
	GO:0031954 positive regulation of protein autophosphorylation	0.8697	7	8.40E‐02

To study the effect of sex, we compared pathways differentially regulated in MCs versus FCs following differentiation. We observed that several pathways related to lipid metabolisms, such as the triglyceride catabolic process, pathway related to the regulation of sequestering of triglycerides, palmitoyltransferase complex pathway, and triglyceride lipase activity pathway, were downregulated males following preadipocyte differentiation, which were absent in females (Table 5).

To study the effect of growth restriction in males and females, we compared the pathways altered in these two study groups. We observed that in FFGR, several pathways related to lipid metabolism were downregulated, which was not the case in MFGR. These pathways were regulation of lipolysis in adipocytes, neutral lipid biosynthetic process, acylglycerol biosynthetic process, triglyceride biosynthetic process, and low‐density lipoprotein particle clearance pathway. The pathways that were differentially regulated in MFGR were related to lipid metabolism and fat pad development pathway (Table 5).

### 3.6 Comparison of genes altered with GWAS studies

We compared the genes known to have SNPs and determined if their expression levels were changed in current studies. To identify the genes with known SNPs in obesity, on the GWAS Catalog, we searched using the terms “obesity” and “Body Mass Index”. We identified 275 unique genes with known SNPs with obesity as a mapped trait (Table S15). Then, we compared our 21 common genes involved in preadipocyte differentiation with the observed SNPs and found that Lrrfip1 was common with the GWAS‐identified list of genes. However, we did not examine the presence of SNPs in the current study.

## 4 DISCUSSION

FGR is an important risk factor in metabolic syndrome, including obesity in adult life (Hales & Barker, 2001; Ozanne et al., 2004). Importantly, growth‐restricted fetuses show a sexually dimorphic risk of obesity development (Dearden et al., 2018). Studies demonstrate that female growth‐restricted newborns are more prone to develop obesity as compared to male growth‐restricted newborns (Dearden et al., 2018). The mechanisms are not known, however.

The current study is a continuation of our ongoing exploration of the “fetal programming of obesity” in response to maternal stress leading to FGR. In our previous report, we demonstrated a sexually dimorphic gene expression in the perirenal adipose tissue in male and female growth‐restricted fetuses as compared to those from normal weight control (Blomberg et al., 2023). Also, we demonstrated that the cellular density was significantly lower in MFGR compared to all other groups. Importantly, there was no difference in the adipocyte size or triglyceride accumulation (Blomberg et al., 2023). Similarly, there was a significant difference between males and females on different premature versus mature adipocyte markers following growth restriction (Blomberg et al., 2023). MFGR demonstrated upregulation of mature adipocyte markers as compared to FFGR. Furthermore, the differentiation ability of perirenal preadipocytes from MFGR was significantly reduced compared to those from MCs, FCs, and growth‐restricted females (Blomberg et al., 2023). Thus, there appears to be genetic programming that reduces the risk of growth‐restricted males from developing obesity compared to growth‐restricted females. In the current study, we observed that with the differentiation of preadipocytes to mature adipocytes, FGR females have similar changes in gene expression to those of control animals and those shown by other studies on mice and humans (Table 4). However, several of these genes were not altered in MFGR with differentiation of preadipocytes to adipocytes.

The 6 genes (Dgat2, Fabp4, Lipe, Lrrfip1, Spred3, and Thrsp), which were not altered in MFGR as compared to all other study groups, could be responsible for this reduction in the differentiation potential of preadipocytes from MFGR, which may be responsible for the reduced incidence of obesity compared to FFGR. In particular, FABP4 was not altered in MFGR, which has been shown to increase significantly with preadipocyte differentiation and adipogenesis (Ramirez et al., 2020; Wang et al., 2018). Similarly, Dgat2 (diacylglycerol O‐acyltransferase 2), which regulates the synthesis of triacylglycerols, was not altered in MFGR. Studies have shown that Dgat2 during preadipocyte differentiation promotes lipid droplet formation, triglyceride content, and expression of other adipogenesis genes (Guo et al., 2023). Another important gene, which was not differentially altered in MFGR perirenal tissue, was the hormone‐sensitive lipase gene LIPE. This gene is known to play a sexually dimorphic role in obesity (Jocken et al., 2008; Roepstorff et al., 2006) and may play a role in reducing the risk of obesity in growth‐restricted males as compared to females. Another important gene that could provide immunity to MFGR from the future development of obesity is LRRFIP1. This gene was altered in all groups except MFGR perirenal preadipocytes, which shows limited differentiation potential. A GWAS study demonstrated that a single‐nucleotide polymorphism (SNP) in the LRRFIP1 gene was associated with abdominal adiposity (Plourde et al., 2013). We also observed that SPRED3 (Sprouty‐related EVH1‐domain‐containing protein gene, downregulated in FFGR, was not altered in MFGR preadipocytes following differentiation. Studies have shown that SPRED gene knockout increases weight gain and obesity (Ohkura et al., 2019). Additionally, the Thrsp gene is upregulated in all groups except MFGR and is known to induce lipogenesis and diet‐induced obesity (Anderson et al., 2009). Overall, there appears to be programming in growth‐restricted male fetuses that prevent the development of obesity in adulthood. These genes also provide valid targets for reducing obesity.

In this study, we used ambient hyperthermia to produce placental insufficiency and restrict in‐utero fetal growth as described previously (Blomberg et al., 2023). The ambient hyperthermia model is based on the observation that lambs born in the summer have lower birth weights and higher mortality rates than lambs born in cooler seasons (Heinzen et al., 2023). We have shown that ambient hyperthermia to 40°C (104° Fahrenheit) produced significant FGR in males and females (Blomberg et al., 2023). Of note, despite significant FGR and reduction in body weight, the relative perirenal fat content to body weight was maintained in these animals (Blomberg et al., 2023). Also, the fetal mortality following ambient hyperthermia was ~20%. In this model of FGR, we exposed pregnant ewes to elevated ambient temperatures (40°C for 12 h; 35°C for 12 h; dew point 22°C) from 38 ± 1 to 87 ± 1 days of gestation (total gestation in sheep is ~145 days). Control fetuses are from ewes maintained at 22 ± 1°C and pair‐fed to the hyperthermic ewe group's average ad libitum feed intake. Thus, the FGR effect observed is not because of low food intake. All ewes are given ad libitum access to water and salt. Exposure to warm ambient temperatures during mid‐gestation diverts a significant amount of cardiac output towards the skin and creates a model of placental insufficiency, which leads to fetuses with substantial growth restriction in the near term (Bell et al., 1987; Chen et al., 2010; Galan et al., 1999; Leos et al., 2010; Limesand & Rozance, 2017; Regnault et al., 2002). Terminal studies show that reductions in placental mass precede declines in fetal weight (Limesand & Rozance, 2017; Macko et al., 2013). From this model, we know that progressive FGR is a causal outcome of placental insufficiency, and the most substantial reductions in fetal growth occur during the last third of gestation when the fetal growth rate is at its highest (Bell et al., 1987). The physiological characteristics of this FGR fetal sheep model parallel those reported for human FGR fetuses and other sheep models of placental restriction (Charlton & Johengen, 1985; Robinson et al., 1979; Wallace et al., 2005), which indicates they are common for placental insufficiency and not imposed by heat stress directly. Furthermore, in this reliable sheep model of placental insufficiency‐induced FGR, the development and progression of growth restriction parallel observations for human FGR fetuses, which is rarely diagnosed before 24 weeks of gestation (Ferrazzi et al., 2002). Currently, the causes of differential programming of genes in males and females are unknown. However, recent studies suggest that placental efficiency is crucial in mediating these sex‐specific developmental outcomes (Clifton, 2010; Eriksson et al., 2010). However, we did not observe any changes in the placental weights between the placenta associated with male and FFGR. However, there may be changes at molecular levels, which requires further investigation.

Irrespective of the animal model used, our detailed analysis demonstrated a group of 21 genes (14 upregulated and 7 downregulated in all groups) differentially regulated in the three species (mouse, human, and sheep), both sexes, three different age groups (embryonic, fetal, and adult), and adipose tissues from different sites (pancreatic, subcutaneous inguinal, and perirenal) in untreated controls. Of note, FABP4 showed a maximum increase of more than 500‐fold following differentiation. FABP4 knockout mice and other studies have demonstrated its important role in adipogenesis and insulin resistance (Syamsunarno et al., 2014). However, there are other isoforms of this Fatty acid‐binding protein, which makes this gene dispensable in the case of knockout mice (Makkar et al., 2014). Similarly, genes such as Ascl1, Cebpa, Plin, Lipe, and adipoq, known markers of mature adipocytes, were significantly upregulated following the differentiation of preadipocytes to mature adipocytes. Furthermore, on examining the common 21 genes (Table 4) with known SNPs associated with obesity in the GWAS (Table S15). We observed that Lrrfip1 is common in both GWAS and the set of genes differentially regulated in humans, mice, and sheep. Although the GWAS study showed the association of Lrrfip1 with adiposity, its role, and mechanism in preadipocyte differentiation require further investigation.

## 5 CONCLUSION

The present study demonstrates that with growth restriction, preadipocytes in male fetuses are differentially programmed than in female fetuses, which may be responsible for the lower incidence of obesity observed in males than in females. Specifically, the present study demonstrates that six genes (Dgat2, Fabp4, Lipe, Lrrfip1, Spred3, and Thrsp) were regulated in a sexually dimorphic manner in males as compared to females. These genes may be responsible for reduced differentiation potential and obesity in FGR males compared to FGR females. Another important finding of the present study is that Lrrfip1, associated with obesity, was upregulated with FGR and requires further investigation. The present study also provides a set of genes involved in differentiation, common in several species, age groups, and preadipocytes from different sites. The molecular mechanisms or pathways these genes are involved in are currently unknown and shall be investigated.

## AUTHOR CONTRIBUTIONS

MG—Data analysis and drafting the manuscript; RILR—Obtaining tissue from sheep and preadipocyte isolation; SWL—Creating placental insufficiency model and sheep surgeries; RG—conceived the idea, supervised the research, drawing conclusions, and final manuscript preparation.

## FUNDING INFORMATION

Support was provided by the 2021 University of Arizona Office for Research, Innovation and Impact Core Facilities Pilot Program and National Institute of Health Grant Award 5R03HD108425 to RG.

## ETHICS STATEMENT

All the studies were conducted in accordance with NIH, USDA, and AAALAS guidelines and with the approval of the Institutional Animal Care and Use Committee (IACUC) of the University of Arizona.

## Supporting information


Figure S1.

Table S1.

Table S2.

Table S3.

Table S4.

Table S5.

Table S6.

Table S7.

Table S8.

**Table S9.**.

Table S10.

Table S11.

Table S12.

Table S13.

Table S14.

Table S15.

## References

[phy270143-bib-0001] Anderson, G. W. , Zhu, Q. , Metkowski, J. , Stack, M. J. , Gopinath, S. , & Mariash, C. N. (2009). The Thrsp null mouse (Thrsp(tm1cnm)) and diet‐induced obesity. Molecular and Cellular Endocrinology, 302(1), 99–107. 10.1016/j.mce.2009.01.005 19356628 PMC2671690

[phy270143-bib-0002] Bell, A. W. , Wilkening, R. B. , & Meschia, G. (1987). Some aspects of placental function in chronically heat‐stressed ewes. Journal of Developmental Physiology, 9(1), 17–29. 3559063

[phy270143-bib-0003] Blomberg, J. , Luna Ramirez, R. I. , Goyal, D. , Limesand, S. W. , & Goyal, R. (2023). Sexual dimorphic gene expression profile of perirenal adipose tissue in ovine fetuses with growth restriction. Frontiers in Physiology, 14, 1179288. 10.3389/fphys.2023.1179288 37601643 PMC10437077

[phy270143-bib-0004] Charlton, V. , & Johengen, M. (1985). Effects of intrauterine nutritional supplementation on fetal growth retardation. Biology of the Neonate, 48(3), 125–142. 10.1159/000242164 3931703

[phy270143-bib-0005] Chen, X. , Fahy, A. L. , Green, A. S. , Anderson, M. J. , Rhoads, R. P. , & Limesand, S. W. (2010). beta2‐adrenergic receptor desensitization in perirenal adipose tissue in fetuses and lambs with placental insufficiency‐induced intrauterine growth restriction. The Journal of Physiology, 588, 3539–3549. 10.1113/jphysiol.2010.192310 20643771 PMC2988517

[phy270143-bib-0006] Clifton, V. L. (2010). Review: Sex and the human placenta: Mediating differential strategies of fetal growth and survival. Placenta, 31, S33–S39. 10.1016/j.placenta.2009.11.010 20004469

[phy270143-bib-0007] Crume, T. L. , Scherzinger, A. , Stamm, E. , McDuffie, R. , Bischoff, K. J. , Hamman, R. F. , & Dabelea, D. (2014). The long‐term impact of intrauterine growth restriction in a diverse U.S. cohort of children: The EPOCH study. Obesity (Silver Spring), 22(2), 608–615. 10.1002/oby.20565 23836432 PMC4437590

[phy270143-bib-0008] de Onis, M. , Blossner, M. , & Villar, J. (1998). Levels and patterns of intrauterine growth retardation in developing countries. European Journal of Clinical Nutrition, 52(1), S5–S15. PubMed PMID: 9511014. 9511014

[phy270143-bib-0009] Dearden, L. , Bouret, S. G. , & Ozanne, S. E. (2018). Sex and gender differences in developmental programming of metabolism. Molecular Metabolism, 15, 8–19. 10.1016/j.molmet.2018.04.007 29773464 PMC6066743

[phy270143-bib-0010] Eriksson, J. G. , Kajantie, E. , Osmond, C. , Thornburg, K. , & Barker, D. J. (2010). Boys live dangerously in the womb. American Journal of Human Biology, 22(3), 330–335. 10.1002/ajhb.20995 19844898 PMC3923652

[phy270143-bib-0011] Ferrazzi, E. , Bozzo, M. , Rigano, S. , Bellotti, M. , Morabito, A. , Pardi, G. , Battaglia, F. C. , & Galan, H. L. (2002). Temporal sequence of abnormal Doppler changes in the peripheral and central circulatory systems of the severely growth‐restricted fetus. Ultrasound in Obstetrics & Gynecology, 19(2), 140–146. 10.1046/j.0960-7692.2002.00627.x 11876805

[phy270143-bib-0012] Galan, H. L. , Hussey, M. J. , Barbera, A. , Ferrazzi, E. , Chung, M. , Hobbins, J. C. , & Battaglia, F. C. (1999). Relationship of fetal growth to duration of heat stress in an ovine model of placental insufficiency. American Journal of Obstetrics and Gynecology, 180(5), 1278–1282. 10.1016/s0002-9378(99)70629-0 10329890

[phy270143-bib-0013] Ge, S. X. , Son, E. W. , & Yao, R. (2018). iDEP: An integrated web application for differential expression and pathway analysis of RNA‐Seq data. BMC Bioinformatics, 19(1), 534. 10.1186/s12859-018-2486-6 30567491 PMC6299935

[phy270143-bib-0014] Goyal, R. , & Longo, L. (2013). Maternal protein deprivation: Sexually dimorphic programming of hypertension in the mouse. Hypertension Research, 36(1), 29–35. 10.1038/hr.2012.129 PubMed PMID: 22932874. 22932874

[phy270143-bib-0015] Goyal, R. , Van‐Wickle, J. , Goyal, D. , & Longo, L. (2015). Antenatal maternal low protein diet: ACE‐2 in the mouse lung and sexually dimorphic programming of hypertension. BMC Physiology, 15(1), 2. 10.1186/s12899-015-0016-6 PubMed PMID: 25971747. 25971747 PMC4430899

[phy270143-bib-0016] Guo, P. P. , Jin, X. , Zhang, J. F. , Li, Q. , Yan, C. G. , & Li, X. Z. (2023). Overexpression of DGAT2 regulates the differentiation of bovine Preadipocytes. Animals (Basel), 13(7), 1–19. 10.3390/ani13071195 PMID: 37048451. PMC10093762 37048451

[phy270143-bib-0017] Hales, C. N. , & Barker, D. J. (2001). The thrifty phenotype hypothesis. British Medical Bulletin, 60, 5–20. PubMed PMID: 11809615. 11809615 10.1093/bmb/60.1.5

[phy270143-bib-0018] Hanson, M. A. , & Gluckman, P. D. (2008). Developmental origins of health and disease: New insights. Basic Clinical and Pharmacol Toxicology, 102(2), 90–93. 10.1111/j.1742-7843.2007.00186.x PubMed PMID: 18226060. 18226060

[phy270143-bib-0019] Heinzen, B. C. , Weber, S. H. , Maia, D. , & Sotomaior, C. S. (2023). Productive performance of lambs born in different seasons of the year. Open Vet J, 13(7), 932–941. 10.5455/OVJ.2023.v13.i7.13 37614728 PMC10443829

[phy270143-bib-0020] Jocken, J. W. , Blaak, E. E. , van der Kallen, C. J. , van Baak, M. A. , & Saris, W. H. (2008). Blunted beta‐adrenoceptor‐mediated fat oxidation in overweight subjects: A role for the hormone‐sensitive lipase gene. Metabolism, 57(3), 326–332. 10.1016/j.metabol.2007.10.006 PubMed PMID: 18249203. 18249203

[phy270143-bib-0021] Kanehisa, M. , & Goto, S. (2000). KEGG: Kyoto encyclopedia of genes and genomes. Nucleic Acids Research, 28(1), 27–30. 10.1093/nar/28.1.27 10592173 PMC102409

[phy270143-bib-0022] Kulenkampff, E. , & Wolfrum, C. (2019). Proliferation of nutrition sensing preadipocytes upon short term HFD feeding. Adipocytes, 8(1), 16–25. 10.1080/21623945.2018.1521229 PMC6768278 30269635

[phy270143-bib-0023] Leos, R. A. , Anderson, M. J. , Chen, X. , Pugmire, J. , Anderson, K. A. , & Limesand, S. W. (2010). Chronic exposure to elevated norepinephrine suppresses insulin secretion in fetal sheep with placental insufficiency and intrauterine growth restriction. American Journal of Physiology Endocrinology and Metabolism, 298(4), E770–E778. 10.1152/ajpendo.00494.2009 20086198 PMC2853210

[phy270143-bib-0024] Limesand, S. W. , & Rozance, P. J. (2017). Fetal adaptations in insulin secretion result from high catecholamines during placental insufficiency. The Journal of Physiology, 595(15), 5103–5113. 10.1113/JP273324 28194805 PMC5538202

[phy270143-bib-0025] Luo, W. , Friedman, M. S. , Shedden, K. , Hankenson, K. D. , & Woolf, P. J. (2009). GAGE: Generally applicable gene set enrichment for pathway analysis. BMC Bioinformatics, 10, 161. 10.1186/1471-2105-10-161 19473525 PMC2696452

[phy270143-bib-0026] Macko, A. R. , Yates, D. T. , Chen, X. , Green, A. S. , Kelly, A. C. , Brown, L. D. , & Limesand, S. W. (2013). Elevated plasma norepinephrine inhibits insulin secretion, but adrenergic blockade reveals enhanced beta‐cell responsiveness in an ovine model of placental insufficiency at 0.7 of gestation. Journal of Developmental Origins of Health and Disease, 4(5), 402–410. 10.1017/S2040174413000093 24358443 PMC3864835

[phy270143-bib-0027] Makkar, A. , Mishima, T. , Chang, G. , Scifres, C. , & Sadovsky, Y. (2014). Fatty acid binding protein‐4 is expressed in the mouse placental labyrinth, yet is dispensable for placental triglyceride accumulation and fetal growth. Placenta, 35(10), 802–807. 10.1016/j.placenta.2014.07.008 25096952 PMC4170794

[phy270143-bib-0028] Ohkura, T. , Yoshimura, T. , Fujisawa, M. , Ohara, T. , Marutani, R. , Usami, K. , & Matsukawa, A. (2019). Spred2 regulates high fat diet‐induced adipose tissue inflammation, and metabolic abnormalities in mice. Frontiers in Immunology, 10, 17. 10.3389/fimmu.2019.00017 30723473 PMC6349710

[phy270143-bib-0029] Ozanne, S. E. , Fernandez‐Twinn, D. , & Hales, C. N. (2004). Fetal growth and adult diseases. Seminars in Perinatology, 28(1), 81–87. Epub 2004/04/03. 10.1053/j.semperi.2003.10.015 15058905

[phy270143-bib-0030] Patro, R. , Duggal, G. , Love, M. I. , Irizarry, R. A. , & Kingsford, C. (2017). Salmon provides fast and bias‐aware quantification of transcript expression. Nature Methods, 14(4), 417–419. 10.1038/nmeth.4197 28263959 PMC5600148

[phy270143-bib-0031] Plourde, M. , Vohl, M. C. , Bellis, C. , Carless, M. , Dyer, T. , Dolley, G. , Marette, A. , Despres, J. P. , Bouchard, C. , Blangero, J. , & Perusse, L. (2013). A variant in the LRRFIP1 gene is associated with adiposity and inflammation. Obesity (Silver Spring), 21(1), 185–192. 10.1002/oby.20242 23505185

[phy270143-bib-0032] Ramirez, A. K. , Dankel, S. N. , Rastegarpanah, B. , Cai, W. , Xue, R. , Crovella, M. , Tseng, Y. H. , Kahn, C. R. , & Kasif, S. (2020). Single‐cell transcriptional networks in differentiating preadipocytes suggest drivers associated with tissue heterogeneity. Nature Communications, 11, 2117. 10.1038/s41467-020-16019-9. PubMed PMID: 32355218; PMCID: PMC7192917. PMC7192917 32355218

[phy270143-bib-0033] Reece, E. (2008). Perspectives on obesity, pregnancy and birth outcomes in the United States: The scope of the problem. American Journal of Obstetrics and Gynecology, 198(1), 23–27. 10.1016/j.ajog.2007.06.076 PubMed PMID: 18166298. 18166298

[phy270143-bib-0034] Regnault, T. R. , Orbus, R. J. , de Vrijer, B. , Davidsen, M. L. , Galan, H. L. , Wilkening, R. B. , & Anthony, R. V. (2002). Placental expression of VEGF, PlGF and their receptors in a model of placental insufficiency‐intrauterine growth restriction (PI‐IUGR). Placenta, 23(2–3), 132–144. Epub 2002/04/12. 10.1053/plac.2001.0757 11945079

[phy270143-bib-0035] Robinson, J. S. , Kingston, E. J. , Jones, C. T. , & Thorburn, G. D. (1979). Studies on experimental growth retardation in sheep. The effect of removal of a endometrial caruncles on fetal size and metabolism. Journal of Developmental Physiology, 1(5), 379–398. Epub 1979/10/01. 45373

[phy270143-bib-0036] Roepstorff, C. , Donsmark, M. , Thiele, M. , Vistisen, B. , Stewart, G. , Vissing, K. , Schjerling, P. , Hardie, D. G. , Galbo, H. , & Kiens, B. (2006). Sex differences in hormone‐sensitive lipase expression, activity, and phosphorylation in skeletal muscle at rest and during exercise. American Journal of Physiology. Endocrinology and Metabolism, 291(5), E1106–E1114. 10.1152/ajpendo.00097.2006 16822962

[phy270143-bib-0037] Subramanian, A. , Tamayo, P. , Mootha, V. K. , Mukherjee, S. , Ebert, B. L. , Gillette, M. A. , Paulovich, A. , Pomeroy, S. L. , Golub, T. R. , Lander, E. S. , & Mesirov, J. P. (2005). Gene set enrichment analysis: A knowledge‐based approach for interpreting genome‐wide expression profiles. Proceedings of the National Academy of Sciences of the United States of America, 102(43), 15545–15550. 10.1073/pnas.0506580102 16199517 PMC1239896

[phy270143-bib-0038] Syamsunarno, M. R. , Iso, T. , Yamaguchi, A. , Hanaoka, H. , Putri, M. , Obokata, M. , Sunaga, H. , Koitabashi, N. , Matsui, H. , Maeda, K. , Endo, K. , Tsushima, Y. , Yokoyama, T. , & Kurabayashi, M. (2014). Fatty acid binding protein 4 and 5 play a crucial role in thermogenesis under the conditions of fasting and cold stress. PLoS One, 9(6), es90825. 10.1371/journal.pone.0090825 PMC3946242 24603714

[phy270143-bib-0039] Wallace, J. M. , Regnault, T. R. , Limesand, S. W. , Hay, W. W., Jr. , & Anthony, R. V. (2005). Investigating the causes of low birth weight in contrasting ovine paradigms. The Journal of Physiology, 565, 19–26. 10.1113/jphysiol.2004.082032 15774527 PMC1464509

[phy270143-bib-0040] Wang, S. , Zhang, Y. , Xu, Q. , Yuan, X. , Dai, W. , Shen, X. , Wang, Z. , Chang, G. , Wang, Z. , & Chen, G. (2018). The differentiation of preadipocytes and gene expression related to adipogenesis in ducks (Anas platyrhynchos). PLoS One, 13, e0196371. 10.1371/journal.pone.0196371 29771917 PMC5957414

