# Supplementary figures and images for: Intrauterine fetal growth restriction in sheep leads to sexually dimorphic programming of Preadipocytes' differentiation potential
Source: Physiol Rep. 2024 Dec 3;12(23):e70143. doi: 10.14814/phy2.70143 (PMC11614549; doi:10.14814/phy2.70143)

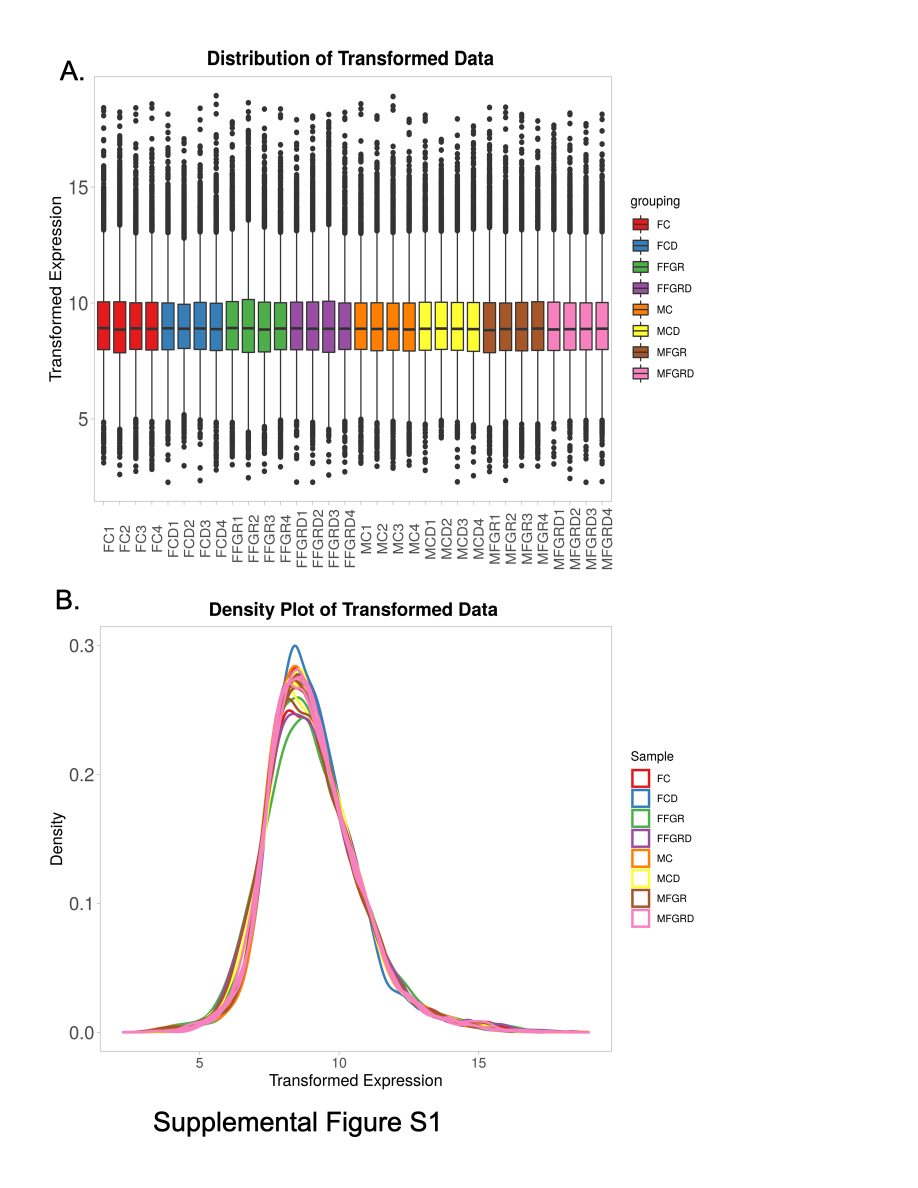

Supplement: Supplementary file 1 — Figure S1. [file PHY2-12-e70143-s014.tif]
